# Epidemiology of and Genetic Factors Associated with *Acanthamoeba* Keratitis

**DOI:** 10.3390/pathogens13020142

**Published:** 2024-02-04

**Authors:** Muhammad Ilyas, Fiona Stapleton, Mark D. P. Willcox, Fiona Henriquez, Hari Kumar Peguda, Binod Rayamajhee, Tasbiha Zahid, Constantinos Petsoglou, Nicole A. Carnt

**Affiliations:** 1 Primary & Secondary Healthcare Department, Punjab 54000, Pakistan; ilyas.fccu@gmail.com (M.I.);; 2 School of Optometry and Vision Science, University of NSW, Sydney, NSW 2052, Australiah.peguda@unsw.edu.au (H.K.P.);; 3 School of Health and Life Sciences, The University of the West of Scotland, Glasgow G72 0LH, UK; 4 Save Sight Institute, The University of Sydney, Sydney, NSW 2000, Australia; 5 Centre for Vision Research, Westmead Institute for Medical Research, Sydney, NSW 2145, Australia

**Keywords:** *Acanthamoeba*, cornea, keratitis, contact lens, pathophysiology, immunology, microbiology

## Abstract

*Acanthamoeba* keratitis (AK) is a severe, rare protozoal infection of the cornea. *Acanthamoeba* can survive in diverse habitats and at extreme temperatures. AK is mostly seen in contact lens wearers whose lenses have become contaminated or who have a history of water exposure, and in those without contact lens wear who have experienced recent eye trauma involving contaminated soil or water. Infection usually results in severe eye pain, photophobia, inflammation, and corneal epithelial defects. The pathophysiology of this infection is multifactorial, including the production of cytotoxic proteases by *Acanthamoeba* that degrades the corneal epithelial basement membrane and induces the death of ocular surface cells, resulting in degradation of the collagen-rich corneal stroma. AK can be prevented by avoiding risk factors, which includes avoiding water contact, such as swimming or showering in contact lenses, and wearing protective goggles when working on the land. AK is mostly treated with an antimicrobial therapy of biguanides alone or in combination with diaminidines, although the commercial availability of these medicines is variable. Other than anti-amoeba therapies, targeting host immune pathways in *Acanthamoeba* disease may lead to the development of vaccines or antibody therapeutics which could transform the management of AK.

## 1. Introduction

*Acanthamoeba* spp. are common free-living amoebae that are present in a diverse range of habitats, including seawater, swimming pools, tap water, hot springs, soil, dust, and even the nasal mucosa of asymptomatic individuals [1,2,3,4,5,6,7]. Pathogenic strains of *Acanthamoeba* can cause severe, life-threatening infections in immunocompromised individuals [8], and they can also lead to serious blindness arising from *Acanthamoeba* keratitis (AK) [9,10]. AK is a less-known corneal infection first reported in 1974 by Nagington and others [11]. Due to its masquerading symptoms, AK is commonly misdiagnosed as viral or bacterial keratitis, resulting in delayed diagnosis and inappropriate treatment [12,13]. Consequently, AK often results in significant loss of sight even after prolonged treatment. This review article will describe the characteristics of *Acanthamoeba* keratitis, including aspects of its epidemiology, disease mechanism, risk behaviors, and preventive measures. The review involved intensive research across multiple databases, including PubMed, Google Scholar, ScienceDirect, and Web of Science, to gather relevant scientific articles.

## 2. Epidemiology

The main risk factor for AK is contact lens (CL) wear, especially in high-income countries where more than 90% of AK patients have a history of contact lens wear [14]. Other factors such as eye trauma, exposure to contaminated water, or exposure to foreign agents like dirt or algae also increase the risk of AK [15,16,17,18]. Studies have also reported increasing non-contact-lens-related AK cases, especially in low–middle-income countries where contact lens wear is less prevalent and AK is mostly associated with corneal injury and exposure to contaminated soil or water [19,20]. While AK is usually a unilateral eye infection, it can also infect both eyes [21,22]. The association between the use of contact lenses and the high incidence of AK was initially documented in 1984 [23]. A study observed a low global incidence of AK from 1980 to 1990, at approximately one to two cases per million contact lens wearers [24]. As the number of contact lens wearers increased, the burden of disease similarly rose [25].

In the United States, the incidence of AK is estimated to be 1 to 2 new cases per 1 million contact lens wearers annually [26]; approximately 17% of the U.S. adult population wears contact lenses [27], so with a population of 340 million people, there may be up to 60 cases per year. Epidemiological studies show that the incidence of AK may have increased in recent years in high-income countries [28,29,30]. Recent outbreaks have been linked to the use of multi-purpose solutions among soft contact lens users [13,31]. A study conducted in the state of Iowa reported that the annual average of new AK cases saw a continuous rise, climbing from 2.9 cases between 2002 and 2009 to 6.5 cases from 2010 to 2017 [17]. Another study involving 13 eye centers around the United States found a sharp and sudden surge in AK cases from 2004 to 2007. The yearly diagnosis count escalated from 22 cases in 1999 to 43 cases in 2003, eventually reaching 170 cases per year by 2007. This outbreak was linked to the use of the Complete Moisture Plus contact lens disinfecting solution [32]. Whilst there was an initial decline in the incidence of AK when this contact lens disinfecting solution was withdrawn from the market, outbreaks have continued, and it is evident that the increase in the number of cases of AK has increased by several orders compared with the period before 2004 [33]. The number of cases seen at Moorfields Eye Hospital in London during the period from 2011 to 2014 (36 to 65 cases per year) was approximately two- to three-fold higher than during the years 2004 to 2010 (15 to 23 cases per year) [13]. The increase in cases was primarily attributed to the use of an Oxipol disinfecting solution for contact lenses. This disinfecting solution has also now been removed from sale worldwide. Another study conducted in Australia at a prominent referral center in Sydney found that the average annual case count from 2002 to 2016 was 50% higher in the years following 2007 compared with the years preceding it. Notably, the highest numbers of cases were documented in the years 2007 and 2014 [34].

## 3. Classification

The classification of *Acanthamoeba* in 1977 was based on cyst morphology [35]. Using this classification system, at least 31 species of *Acanthamoeba* were identified, which were broadly divided into three major groups [36]. Group 1 has five species characterized by cysts equal to or larger than 18 µm [37]. Group 2, the most prevalent, has 17 species, including several pathogenic species [36,38]. Group 3 has nine species with smaller cysts with less distinct outer walls [37,39]. However, as the morphology of *Acanthamoeba* cysts can be influenced by the growth medium [40], there was a clear need for a more scientifically robust classification method [41,42]. Therefore, the taxonomic status of *Acanthamoeba* has evolved through the use of molecular techniques. DNA sequences have provided new insights into the species designation within Acanthamoeba, shedding light on its pathogenic potential, its evolutionary lineages, and alternative approaches to disease treatment [43].

The work on molecular-based classification began in 1996 when *Acanthamoeba* was classified using the whole gene sequence of nuclear small subunit 18S ribosomal RNA (Rns) [44]. This system currently classifies *Acanthamoeba* into 23 genotypes (T1–T23), encompassing all currently known *Acanthamoeba* isolates to date shown in Table 1. This method was further modified by targeting smaller gene segments, such as 280 base-pair (bp)-long highly variable diagnostic fragment 3 (DF3) in the 464 bp-long *Acanthamoeba*-specific amplimer (ASA.S1) [45]. This technology has facilitated the subdivision of T2 into two further groups named T2a and T2b [46]. Moreover, T4 has recently been subclassified into eight distinct groups named T4A, T4B, T4C, T4D, T4E, T4F, T4G/T4Neff, and T4H [38]. Whilst the DF3 fragment has helped in epidemiological and phylogenetic studies, more studies are required to examine its limitations. Therefore, the use of full-length sequences of *Acanthamoeba* 18S rRNA is strongly recommended [47,48].

Different genotypes of *Acanthamoeba* are associated with different pathogenicity, disease severity, and clinical presentation. Understanding the genotypes may assist in better clinical management of AK [49]. The T4 genotype is the most prevalent *Acanthamoeba* genotype in nature [50], and it is identified in the majority of human infections, particularly those associated with AK [51,52,53]. Most of the *Acanthamoeba* isolates identified from patients with the most severe infections are also from the T4 genotype [54], especially the T4A sub-genotype, followed by the T3 genotype [51]. Other less common genotypes, T2, T5, T6, T8, T9, T10, T11, T12, T13, and T15, have also been recovered from AK patients [55,56,57]. One study has argued that genotype T5 is not inherently a human pathogen but has recently become more associated with AK due to increased contact lens usage [53].

**Table 1:** The traditional classification of *Acanthamoeba*.

Morphological Group	Species	Genotype	Strain Type	Reference
**Group-I**	*A. astronyxis*	T7	ATCC 30137	[58]
	*A. comandoni*	T9	ATCC 30135	[59]
	*A. echinulate*	T4	ATCC 50239	[35]
	*A. tubiashi*	T8	ATCC 30867	[60]
	*A. byersii*	T18	PRA- 411	[61]
**Group-II**	*A. castellanii*	T4	ATCC 50374 = 30011	[62]
	*A. terricola*	T4	ATCC 30134	[63]
	*A. gigantean*	–	ATCC 50670	[64]
	*A. polyphaga*	T4	ATCC 30871	[65]
	*A. griffinii*	T3	ATCC 30731	[66]
	*A. rhysodes*	T4	ATCC 30973	[67]
	*A. diuionensis*	T4	ATCC 50238	[35]
	*A. lugdunensis*	T4	ATCC 50240	[35]
	*A. quina*	T4	ATCC 50241	[35]
	*A. paradiuionensis*	T4	ATCC 50251	[35]
	*A. mauritaniensis*	T4	ATCC 50253	[35]
	*A. triangularis*	T4	ATCC 50254	[35]
	*A. hatchetti*	T11	ATCC 30730	[68]
	*A. stevensoni*	T11	ATCC 50388	[69]
	*A. pearcei*	T3	ATCC 50435	[70]
	*A.micheli*	T19	BRO2-T19	[47]
	*A. pyriformis*	T21	CCAP 1501/19	[71]
**Group-III**	*A. palestinensis*	T2	ATCC 30870	[72]
	*A. culbertsoni*	T10	ATCC 30171	[67]
	*A. pustulosa*	T2	ATCC 50252	[35]
	*A. royreba*	T4	ATCC 30884	[73]
	*A. lenticulata*	T5	ATCC 30841	[74]
	*A. healyi*	T12	ATCC 30866	[75]
	*A. jacobsi*	T15	ATCC 30732	[76]
	*A. sohi*	–	Acanthamoeba YM-4	[77]
	* A. bangkokensis *sp.* nov. *	T23	AcW61	[78]

## 4. Life Cycle and Morphology

*Acanthamoeba* species are present almost everywhere, from thermal springs to sub-glacial environments and every conceivable habitat in between [79]. The life cycle of *Acanthamoeba* species consists of two stages. They exist as either motile, vegetative trophozoites or dormant, highly resistant cysts [16,80]. The trophozoite stage dominates when the environmental conditions are favorable for life, such as an abundant supply of water and food, a neutral pH, an optimum temperature (around 30 °C), and an osmolarity ranging between 50 and 80 mOsmol [80]. *Acanthamoeba* trophozoites are heterotrophs that primarily consume bacteria, viruses, yeast, algae, or small organic particles through phagocytosis or pinocytosis, forming food vacuoles within the cytoplasm [81,82]. Trophozoites are oval or irregular in shape and are characterized by their typical spine-shaped pseudopods, now known as acanthopodia that extend from the clear ectoplasm [82]. Apart from aiding in movement and feeding, these acanthopodia also help *Acanthamoeba* trophozoites cause corneal infection by adhering to the surface of a contact lens [83,84].

Under unfavorable environmental conditions, the trophozoites transform into highly resistant, double-walled, quiescent cysts in a process called encystation [85]. This process involves drastic changes in gene expression, which prepares *Acanthamoeba* to acclimatize to the new environment. Cysts have a rigid, double-layered cell wall which enables *Acanthamoeba* to survive in remarkably harsh environmental conditions such as hyperosmolarity, glucose starvation, desiccation, extreme pH and temperature, the presence of chemicals and toxins, and high doses of radiation [80,86,87]. Cysts have survived more than 20 years of storage at room temperature with no water or food source and were still able to develop into viable trophozoites when the environmental conditions became favorable [86,88]. The cystic form of *Acanthamoeba* shows minimal metabolic activity, making it highly resistant to anti-amoebal agents [89]. Due to their resistance to contact lens disinfecting solutions and disinfectants, they can contaminate lens storage cases [15]. Cysts are resistant to fungicides, chlorination, and a range of antimicrobials [90,91]. Cysts can remain dormant within corneal tissues for up to 31 months and then produce a recurrence of keratitis [92].

## 5. Pathogenesis

The pathogenesis of AK occurs in two distinct phases. In the first phase, *Acanthamoeba* attaches to and infiltrates the corneal epithelium, followed by the invasion of the underlying stroma in the secondary phase. In contact-lens-associated AK, *Acanthamoeba* adheres to the back surface of the contact lens, perhaps from contaminated lens disinfecting solutions, lens storage cases, or low-level environmental sources, such as domestic water, and subsequently transfers to the corneal surface. Adhesion is facilitated by acanthopodia in trophozoites [84], but cysts can also adhere to contact lenses [93]. Trophozoites or cysts may be cleared from the ocular surface by tears, the blinking action of the eyes, and the natural immune response of the eyes [94]. However, contact lenses reduce post-lens tear exchange, thereby facilitating attachment to the corneal surface [39]. Additionally, contact lens use may induce microabrasions to the corneal epithelial surface, conceivably making it vulnerable to potential attacks by pathogenic microbes [39].

*Acanthamoeba* produces adhesive proteins, such as mannose-binding proteins (MBPs) and laminin-binding proteins (LBPs), which bind mannosylated glycoproteins and laminin glycoproteins, respectively, on the corneal epithelium [95,96]. Pathogenic strains of *Acanthamoeba* exhibit high levels of MBPs and LBPs [97,98]. In contrast, non-pathogenic strains of *Acanthamoeba* have fewer acanthopodia per cell (<20 acanthopodia/cell compared with >100 acanthopodia/cell in pathogenic strains) and low levels of binding to MBPs and LBPs (Table 2), resulting in a reduced binding capacity to adhere to host cells [24]. Initially, the infection is limited to the epithelium, but *Acanthamoeba* can penetrate the epithelial barrier by direct killing and inducing apoptosis of cells. After the breakdown of the epithelial layer, the disease progresses to the next phase, in which trophozoites attach to the underlying collagenous stroma. *Acanthamoeba* can also produce phospholipases that disrupt the host cell plasma membrane. This results in the loss of cellular integrity, followed by lysis [99,100]. Neuraminidase can target sialic acid in the corneal epithelium, causing damage to the epithelial cells and facilitating *Acanthamoeba* colonization [101,102]. The combined effect of these factors may contribute to the destruction and dissemination of *Acanthamoeba,* which degrades further into the stromal matrix [103,104].

*Acanthamoeba* produces three distinct types of proteases: serine proteases, cysteine proteases, and metalloproteases [39]. Serine proteases are the most abundant and can be found in nearly all *Acanthamoeba* species [105,106]. Serine proteases break down collagen type 1, which is present in the corneal stroma and regulates corneal integrity. Additionally, serine proteases attack and degrade secretory immunoglobulin A (IgA), immunoglobulin G (IgG), fibrinogen, fibronectin, laminin, fibrin, hemoglobin, plasminogen, and bovine serum albumin. Cysteine proteases are implicated in the degradation of host cells [107] and can hydrolyze various proteins including immunoglobulin A (IgA), immunoglobulin G (IgG), hemoglobin, albumin, and fibronectin [108,109]. Metalloproteinases also contribute to the initiation of cytotoxic effects on the host cells. These metalloproteinases can degrade collagen, plasminogen, elastin, casein, and gelatin [110,111]. Trophozoites then feed on keratocytes and organic particles within the stroma, resulting in keratocyte depletion, a robust inflammatory response, and ultimately stromal necrosis [21,22].

AK is often accompanied by exquisite pain. The reason behind the intense pain linked to AK is not fully understood. However, the movement of *Acanthamoeba* toward chemotactic signals might lead to their association with nerve cells in the cornea. Clinically, the disease is characterized by perineural infiltrates [15], which may indicate neurogenic inflammation. Additionally, enzymes such as MIP133 (mannose-induced protein, a serine protease with high cytolytic activity) could play a role in causing damage to the nerves, ultimately contributing to the pain associated with this infection. Advanced disease stages can potentially kill the corneal nerves by sequential activation of caspase-3 and caspase-10 mediated apoptosis, with potential sight-threatening effects [14].

## 6. Innate and Adaptive Host Immune Responses

When *Acanthamoeba* trophozoites attack the ocular surface, they encounter innate immune cells that promptly defend against the initiation of ocular infection [112]. Studies have highlighted the ability of macrophages to kill *Acanthamoeba,* especially when activated by interferon-γ (IFN-γ) [113,114]. Depleting periocular macrophages through the injection of a subconjunctival macrophage-killing drug, clodronate, resulted in a significant worsening of AK in a Chinese hamster model of the disease [112]. Similarly, studies have shown promising evidence regarding the ability of neutrophils to kill *Acanthamoeba* cysts and trophozoites in vitro [115]. In a Chinese hamster model, introducing up to a million *Acanthamoeba* trophozoites into the anterior chamber of the eye resulted in a vigorous neutrophil response that efficiently eradicated the considerable trophozoite inoculum within 15 days following the injection [116]. In a separate animal-based study, the introduction of MIP-2 (an animal homologue of human IL-8), a potent chemotactic attractant for neutrophils, directly into the cornea alleviated AK. Conversely, the injection of anti-MIP-2 inhibited neutrophil migration to the infection site and led to the worsening of the disease [117].

The role of IL-8 is also important in the pathogenesis of AK. In severe cases of AK, IL-8 is expressed at higher concentrations compared with milder cases [118]. This elevated expression is associated with scleritis. IL-8 activates and attracts neutrophils to the sclera, serving as key inflammatory mediators and contributing to the worsening of AK [118]. Unlike most other inflammatory cytokines, IL-8 remains active at the site of inflammation for up to several weeks, leading to sustained inflammation [119]. Additionally, IL-8 is also a part of the Toll-like receptor 4 (TLR-4) cascade, which initiates the cytokine response in AK [119]. The prolonged persistence of IL-8 and other cytokines in the cornea could significantly contribute to ongoing inflammation in AK, which would explain the severe clinical symptoms in affected individuals. Furthermore, IL-8 also promotes angiogenesis, which may contribute to ocular neovascularization, a late complication associated with AK [118].

Serological surveys indicate that 90 to 100% of the asymptomatic population with no history of *Acanthamoeba* infections expresses serum IgG antibodies specific for *Acanthamoeba* [120]. This is not unexpected as cysts are present in air and dust and are inhaled daily. These antibodies, in addition to IgA which is present in the highest concentrations in tears [24], are very crucial in neutralizing cytotoxic effects produced by the amoeba and preventing *Acanthamoeba* adhesion to the host cell by blocking trophozoite movement. IgG may also resist the trophozoite’s progress during the stromal invasion [121]. Furthermore, secretory IgG antibodies produced in response to mannosylated glycoproteins of *Acanthamoeba* are thought to activate the complement system by attaching to Fc receptors on the neutrophils. Once activated, these neutrophils attack and kill trophozoites [115,122]. Similarly, most of the asymptomatic population shows robust T-cell responses to *Acanthamoeba* exposure [14]. The high occurrence of T and B lymphocyte activation in individuals without a history of *Acanthamoeba* infections emphasizes the ubiquitous nature of environmental exposure to *Acanthamoeba*. However, it is important to note that AK is believed to be caused by the transfer of *Acanthamoeba* from a contact lens to the corneal surface. The corneal surface is considered an immune-privileged avascular site, and in this case, *Acanthamoeba* may not elicit rapid antibodies or T-cell responses on the ocular surface [123]. Both humoral and delayed-type responses are produced by systemic immunization with *Acanthamoeba* antigens, but these responses cannot provide protection against ocular infection [123].

Since corneal infection caused by *Acanthamoeba* does not produce immunity, persistence of this infection is frequent. This persistence is due to the capacity of *Acanthamoeba* to avoid the immune response to the infection, thus being able to persist in host tissue for extended periods.Top of Form

## 7. Immune Evasion

*Acanthamoeba* has evolved several mechanisms to evade a host’s immune system and cause persistent infections. This characteristic makes AK a challenging disease to manage with conventional techniques. Whilst the surface antigens of *Acanthamoeba* can elicit a humoral immune response, leading to the production of secretory IgA and serum IgG antibodies, *Acanthamoeba* trophozoites suppress this immune response by secreting serine proteases, which can cleave IgA and IgG [124,125]. There are lower levels of *Acanthamoeba*-specific IgA and IgG antibodies in AK patients than in healthy individuals [120,126]. These decreased levels of antibodies enable *Acanthamoeba* to adhere to the host corneal epithelium and survive in the host for a longer period.

**Figure 1:**
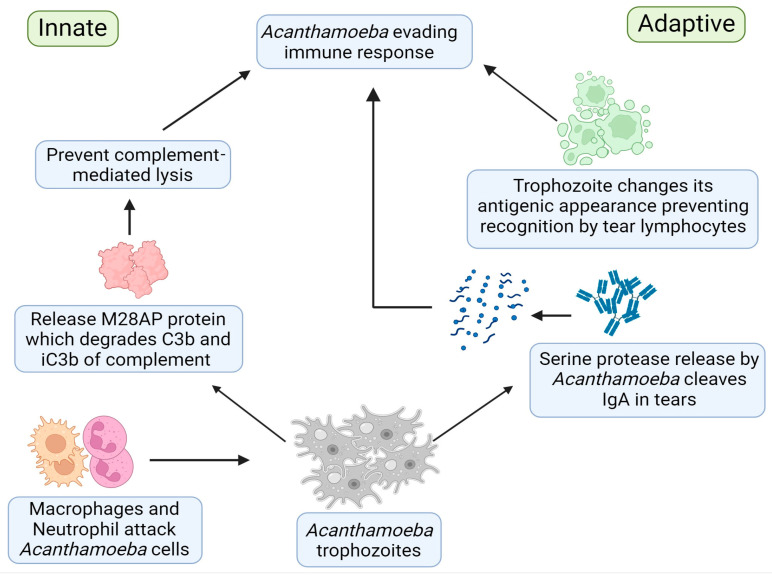
Immune evasion strategies used by *Acanthamoeba*.

A pathological examination of an *Acanthamoeba*-infected cornea revealed the absence of lymphocytes from the AK lesion [127], which suggests that *Acanthamoeba* trophozoites can change their antigenic appearance by masking their surfacing antigens and escape immune recognition from the host immune system (Figure 1) [128]. This mechanism may explain why some individuals fail to recover from *Acanthamoeba* infection [129].

The pathogenic strains of *Acanthamoeba* trophozoites express complement regulatory proteins, which can downgrade complement-mediated lysis, making them less susceptible to the antimicrobial effects of the complement [128]. This resistance to the antimicrobial properties of the complement may explain why animals immunized against *Acanthamoeba* and expressing complement-fixing antibodies are not protected against AK. A systematic analysis has identified a unique protein secreted by *Acanthamoeba* isolates, M28 aminopeptidase (M28AP), which can degrade crucial human complement proteins, such as C3b and iC3b [130]. This characteristic disables the innate arm of the complement system, enhancing the pathogenicity of *Acanthamoeba*. Pathogenic isolates of *Acanthamoeba* have greater resistance to complement compared with non-pathogenic isolates [131,132]. It is important to note that other pathogenic protozoa, like *Naegleria fowleri*, also demonstrate a similar pattern to complement-mediated lysis [133].

*Acanthamoeba* trophozoites undergo encystation within human tissue as a strategy to escape the host immunologic response. This characteristic enables *Acanthamoeba* to resist immune attacks and survive in a hostile host environment. Studies have shown that cysts are less chemoattractant for macrophages and lymphocytes [134]. This characteristic of cysts is attributed to their inert nature and fewer viable receptors for immune cells. Despite successful treatment, cysts can persist in the corneal stroma for several years, where they are less immunogenic and fewer in number than trophozoites [135]. Additionally, the eye’s immunologically privileged nature provides further protection to these residing cysts, preventing recognition and elimination by the adaptive immune system [126,136]. These cysts are a crucial source for the recurrence of corneal infections [135].

## 8. Genetic Variations Associated with the Severity of Disease

Several host genetic variations are associated with severe inflammatory complications (SICs) in AK patients. Single nucleotide polymorphisms (SNPs) in the CXCL8 gene, which encodes IL-8, were significantly associated with protection from SICs [119]. Conversely, SNPs in the TLR-4 gene have been associated with an increased risk of SICs, and SNPs in Th-17-related genes (IL-23R, IL-1β, and IL-22) were also found to be associated with an increased incidence of SICs in AK patients [119]. A study of non-amoebic microbial keratitis found associations between genetic variations in interleukins and disease severity. SNPs in the IL-6 gene were associated with more severe clinical outcomes [137]. Moreover, variations in beta defensin 1 (DEBF1) gene expression were associated with increased susceptibility to contact-lens-related keratitis [138]. SNPs in IL-17F were also possibly associated with more severe outcomes of microbial keratitis [139].

## 9. Diagnosis

*Acanthamoeba* is a rare cause of keratitis, which is why it is often overlooked as a differential diagnosis by clinicians. Early diagnosis of AK is essential to avoid serious damage to the cornea and vision. It is important to have a clinical suspicion of AK in patients with associated risk factors. The different diagnostic techniques have their strengths and limitations. Therefore, multiple diagnostic approaches are used for the diagnosis of AK. In clinical settings, culture is most commonly used along with in vivo confocal microscopy (IVCM) and polymerase chain reaction (PCR).

The culture of *Acanthamoeba* is considered the gold standard and plays an important role in diagnosing suspected cases of AK. It is usually performed with corneal scrapings and gives 100% specificity [140]. However, the sensitivity of the culture is reported to be below 67% [141,142,143] and is influenced by the culture method used [144]. The technique for obtaining the corneal specimen can also impact the diagnostic sensitivity. Corneal biopsy has the highest sensitivity, followed by spatula methods, while specimens obtained with a cotton swab have the lowest sensitivity [145]. The sensitivity of the culture is improved when combined with a direct smear stained with stains like Calcofluor white, which helps identify *Acanthamoeba* cysts [146].

IVCM is a non-invasive diagnostic tool recently employed for AK diagnosis. IVCM has a very high specificity (100%) and a high sensitivity, ranging from 85 to 100% [147,148]. The test also provides rapid results, especially when compared with other diagnostic tools. This technique can detect *Acanthamoeba* cysts only, which makes it limited to detecting AK at later stages of the disease [149]. Moreover, stromal inflammation and edema can cause masking of *Acanthamoeba cysts*, resulting in false-negative results. Apart from diagnosis, IVCM is also used to assess treatment outcomes and examine for residual disease [150].

PCR of corneal scrapings is becoming increasingly popular for diagnosing AK, providing faster results with a more accessible diagnostic modality than culture. PCR offers high specificity (100%), and the sensitivity of a single PCR assay for AK is reported to be approximately 70%, which is higher than some culture-based methods, with the potential to increase up to 93% or more when multiple gene assays are used [142,143]. However, the sensitivity of PCR is reduced in the initial phase of infection, particularly with small specimen sizes and in cases undergoing anti-Acanthamoebal treatment [151]. The strengths and limitations of these diagnostic tools are summarized in Table 3.

## 10. Prevention

Increased awareness regarding the associated risks of poor contact lens hygiene practices is important. Key practices include avoiding contact between tap water and contact lenses by not handling lenses with wet hands, and not wearing contact lenses when showering or swimming. Hand hygiene is important, so washing hands with soap and water and drying them thoroughly before handling contact lenses may help to reduce the risk of corneal infection. The use of self-made saline solutions and expired disinfectants is highly discouraged. Only ISO-certified and FDA-approved disinfecting solutions should be used for cleaning and storing contact lenses. Failure to clean or replace contact lens cases is associated with a higher risk of severe infection.

## 11. Conclusions/Future Directions

AK presents a rare yet serious corneal infection, posing significant challenges in both diagnosis and treatment. The infection is caused by a ubiquitous protozoon *Acanthamoeba* and particularly affects vulnerable groups, notably contact lens wearers and those who have recent eye trauma. AK is often misdiagnosed, resulting in delayed intervention. The host immune response to the infection is crucial for disease eradication, but it simultaneously poses risks such as scarring and vision loss due to increased corneal inflammation. The genetic similarity between *Acanthamoeba* and humans poses a challenge in finding an appropriate treatment method that selectively harms the parasite without causing harm to the human host. In this regard, the development of *Acanthamoeba*-specific IgA vaccine in tears, capable of blocking trophozoite adherence to mannosylated proteins on the corneal epithelium, can be effective in preventing infection.

The ability of *Acanthamoeba* to produce several proteases during infection can be another potential avenue for therapeutic interventions. Identifying the exact molecular mechanisms of how these proteases work may help in developing proteinase inhibitors. Further research is necessary to explore the potential role of protease inhibitors as treatments. *Acanthamoeba* trophozoites can transform into cysts in the cornea, resulting in the recurrence of infection after treatment. Developing treatments that can target distinct morphological structures of cysts may offer better clinical management options. Furthermore, genetic screening for specific SNPs associated with severe inflammatory complications in AK patients could help predict the risk of disease severity, allowing for more personalized and targeted management of the disease.

## Figures and Tables

**Table 2:** Morphological differences between pathogenic and non-pathogenic *Acanthamoeba*.

Characteristics	Pathogenic Amoeba	Non-Pathogenic Amoeba
**Acanthopodia**	Higher number of complex Acanthopodia.	Lower number of less-complex Acanthopodia.
**Adherence to host cells**	Higher number of MBPs and LBPs results in higher affinity for adherence to host cells.	Smaller number of MBPs and LBPs results in lower affinity for adherence to host cells.
**Cyst formation**	Formation of highly resistant cysts with complex outer layers.	Formation of cysts with simpler outer layers, potentially less resistant.
**Motility**	May display increased motility and faster movement.	Generally slower and less dynamic motility.
**Surface features**	Presence of distinctive surface structures, such as ridges or spines.	Surface features are typically smoother and less pronounced.

**Table 3:** Comparison between different methods in the diagnosis of *Acanthamoeba* keratitis.

Method	Strengths	Limitations
Culture	1. Gold standard for AK diagnosis. 2. Easy to perform.	1. Requires 1–2 weeks for a positive result. 2. Influenced by specimen type.
IVCM	1. High specificity and sensitivity. 2. Non-invasive. 3. Instant results.	1. Recognizes cysts only. 2. Expensive and not widely available. 3. User-dependent.
PCR	1. High specificity and sensitivity. 2. Rapid results. 3. Allows genotyping, which helps in disease management and epidemiological studies.	1. False-positive results in presence of dead amoeba. 2. False-negative results during treatment. 3. Reduced sensitivity in the initial phase of infection.

## References

[1] Rivera F., Lares F., Gallegos E., Ramirez E., Bonilla P., Calderon A., Martinez J.J., Rodriguez S., Alcocer J. (1989). Pathogenic amoebae in natural thermal waters of three resorts of Hidalgo, Mexico. Environ. Res..

[2] Rivera F., Lares F., Ramirez E., Bonilla P., Rodriguez S., Labastida A., Ortiz R., Hernandez D. (1991). Pathogenic *Acanthamoeba* isolated during an atmospheric survey in Mexico City. Clin. Infect. Dis..

[3] Michel R., Hauröder-Philippczyk B., Müller K.-D., Weishaar I. (1994). *Acanthamoeba* from human nasal mucosa infected with an obligate intracellular parasite. Eur. J. Protistol..

[4] Tawfeek G.M., Bishara S.A.-H., Sarhan R.M., ElShabrawi Taher E., ElSaady Khayyal A. (2016). Genotypic, physiological, and biochemical characterization of potentially pathogenic *Acanthamoeba* isolated from the environment in Cairo, Egypt. Parasitol. Res..

[5] Lass A., Guerrero M., Li X., Karanis G., Ma L., Karanis P. (2017). Detection of *Acanthamoeba* spp. in water samples collected from natural water reservoirs, sewages, and pharmaceutical factory drains using LAMP and PCR in China. Sci. Total Environ..

[6] Carnt N.A., Subedi D., Lim A.W., Lee R., Mistry P., Badenoch P.R., Kilvington S., Dutta D.J.C., Optometry E. (2020). Prevalence and seasonal variation of Acanthamoeba in domestic tap water in greater Sydney, Australia. Clin. Exp. Optom..

[7] Wopereis D.B., Bazzo M.L., de Macedo J.P., Casara F., Golfeto L., Venancio E., de Oliveira J.G., Rott M.B., Caumo K.S. (2020). Free-living amoebae and their relationship to air quality in hospital environments: Characterization of *Acanthamoeba* spp. obtained from air-conditioning systems. Parasitology.

[8] Sharma G., Kalra S.K., Tejan N., Ghoshal U. (2020). Nanoparticles based therapeutic efficacy against *Acanthamoeba*: Updates and future prospect. Exp. Parasitol..

[9] Pacella E., La Torre G., De Giusti M., Brillante C., Lombardi A.M., Smaldone G., Lenzi T., Pacella F. (2013). Results of case-control studies support the association between contact lens use and *Acanthamoeba* keratitis. Clin. Ophthalmol..

[10] Nwachuku N., Gerba C.P. (2003). Health effects of *Acanthamoeba* spp. and its potential for waterborne transmission. Reviews of Environmental Contamination and Toxicology.

[11] Nagington J., Watson P., Playfair T., McGill J., Jones B., Steele A. (1974). Amoebic infection of the eye. Lancet.

[12] Tu E.Y., Joslin C.E., Sugar J., Shoff M.E., Booton G.C. (2008). Prognostic factors affecting visual outcome in *Acanthamoeba* keratitis. Ophthalmology.

[13] Carnt N., Hoffman J.J., Verma S., Hau S., Radford C.F., Minassian D.C., Dart J.K.G. (2018). *Acanthamoeba* keratitis: Confirmation of the UK outbreak and a prospective case-control study identifying contributing risk factors. Br. J. Ophthalmol..

[14] Niederkorn J.Y., Alizadeh H., Leher H., McCulley J.P. (1999). The pathogenesis of *Acanthamoeba* keratitis. Microbes Infect..

[15] Carnt N., Stapleton F. (2016). Strategies for the prevention of contact lens-related *Acanthamoeba* keratitis: A review. Ophthalmic Physiol. Opt..

[16] Dos Santos D.L., Kwitko S., Marinho D.R., De Araújo B.S., Locatelli C.I., Rott M.B. (2018). *Acanthamoeba* keratitis in Porto Alegre (southern Brazil): 28 cases and risk factors. Parasitol. Res..

[17] Scruggs B.A., Quist T.S., Salinas J.L., Greiner M.A. (2019). Notes from the field: *Acanthamoeba* keratitis cases—Iowa, 2002–2017. Morb. Mortal. Wkly. Rep..

[18] Carnt N.A., Subedi D., Connor S., Kilvington S. (2020). The relationship between environmental sources and the susceptibility of *Acanthamoeba* keratitis in the United Kingdom. PLoS ONE.

[19] Buerano C.C., Trinidad A.D., Fajardo L.S.N., Cua I.Y., Baclig M.O., Natividad F.F. (2014). Isolation of *Acanthamoeba* genotype T4 from a non-contact lens wearer from the Philippines. Trop. Med. Health.

[20] Page M.A., Mathers W.D. (2013). *Acanthamoeba* keratitis: A 12-year experience covering a wide spectrum of presentations, diagnoses, and outcomes. J. Ophtalmol..

[21] Lee W.B., Gotay A. (2010). Bilateral *Acanthamoeba* keratitis in Synergeyes contact lens wear: Clinical and confocal microscopy findings. Eye Contact Lens Sci. Clin. Pract..

[22] Voyatzis G., McElvanney A. (2007). Bilateral *Acanthamoeba* keratitis in an experienced two-weekly disposable contact lens wearer. Eye Contact Lens Sci. Clin. Pract..

[23] Lindsay R.G., Watters G., Johnson R., Ormonde S.E., Snibson G.R. (2007). *Acanthamoeba* keratitis and contact lens wear. Clin. Exp. Optom..

[24] Lorenzo-Morales J., Martín-Navarro C.M., López-Arencibia A., Arnalich-Montiel F., Piñero J.E., Valladares B. (2013). *Acanthamoeba* keratitis: An emerging disease gathering importance worldwide?. Trends Parasitol..

[25] Zhang Y., Xu X., Wei Z., Cao K., Zhang Z., Liang Q. (2023). The global epidemiology and clinical diagnosis of *Acanthamoeba* keratitis. J. Infect. Public Health.

[26] Schaumberg D.A., Snow K.K., Dana M.R. (1998). The epidemic of Acanthamoeba keratitis: Where do we stand?. Cornea.

[27] Cope J.R., Collier S.A., Rao M.M., Chalmers R., Mitchell G.L., Richdale K., Wagner H., Kinoshita B.T., Lam D.Y., Sorbara L. (2015). Contact lens wearer demographics and risk behaviors for contact lens-related eye infections—United States, 2014. Morb. Mortal. Wkly. Rep..

[28] Nielsen S.E., Ivarsen A., Hjortdal J. (2020). Increasing incidence of *Acanthamoeba* keratitis in a large tertiary ophthalmology department from year 1994 to 2018. Acta Ophthalmol..

[29] Randag A.C., Van Rooij J., Van Goor A.T., Verkerk S., Wisse R.P., Saelens I.E., Stoutenbeek R., van Dooren B.T., Cheng Y.Y., Eggink C.A. (2019). The rising incidence of *Acanthamoeba* keratitis: A 7-year nationwide survey and clinical assessment of risk factors and functional outcomes. PLoS ONE.

[30] Graffi S., Peretz A., Jabaly H., Koiefman A., Naftali M. (2013). *Acanthamoeba* keratitis: Study of the 5-year incidence in Israel. Br. J. Ophthalmol..

[31] Joslin C.E., Tu E.Y., Shoff M.E., Booton G.C., Fuerst P.A., McMahon T.T., Anderson R.J., Dworkin M.S., Sugar J., Davis F.G. (2007). The association of contact lens solution use and *Acanthamoeba* keratitis. Arch. Ophthalmol..

[32] Yoder J.S., Verani J., Heidman N., Hoppe-Bauer J., Alfonso E.C., Miller D., Jones D.B., Bruckner D., Langston R., Jeng B.H. (2012). *Acanthamoeba* keratitis: The persistence of cases following a multistate outbreak. Ophthalmic Epidemiol..

[33] Tu E.Y. (2014). Acanthamoeba keratitis: A new normal. Am. J. Ophthalmol..

[34] Höllhumer R., Keay L., Watson S.J. (2020). Acanthamoeba keratitis in Australia: Demographics, associated factors, presentation and outcomes: A 15-year case review. Eye.

[35] Pussard M. (1977). Morphologie de la paroikystique et taxonomie du genre Acanthamoeba (Protozoa, Amoebida). Protistologica.

[36] Wang Y., Jiang L., Zhao Y., Ju X., Wang L., Jin L., Fine R.D., Li M. (2023). Biological characteristics and pathogenicity of *Acanthamoeba*. Front. Microbiol..

[37] Kong H.H. (2009). Molecular phylogeny of *Acanthamoeba*. Korean J. Parasitol..

[38] Corsaro D. (2020). Update on *Acanthamoeba* phylogeny. Parasitol. Res..

[39] de Lacerda A.G., Lira M. (2021). *Acanthamoeba* keratitis: A review of biology, pathophysiology and epidemiology. Ophthalmic Physiol. Opt..

[40] Fuerst F., McAllister P., Nanda A., Wyatt P. (2015). Does energy efficiency matter to home-buyers? An investigation of EPC ratings and transaction prices in England. Energy Econ..

[41] Khan N.A. (2006). *Acanthamoeba*: Biology and increasing importance in human health. FEMS Microbiol. Rev..

[42] Castrillón J.C., Orozco L.P. (2013). *Acanthamoeba* spp. como parásitos patógenos y oportunistas. Rev. Chil. Infectol..

[43] Fuerst P.A., Booton G.C. (2020). Species, sequence types and alleles: Dissecting genetic variation in *Acanthamoeba*. Pathogens.

[44] Gast R.J., Ledee D.R., Fuerst P.A., Byers T.J. (1996). Subgenus systematics of *Acanthamoeba*: Four nuclear 18S rDNA sequence types. J. Eukaryot. Microbiol..

[45] Taher E.E., Méabed E.M., Abdallah I., Wahed W.Y.A. (2018). *Acanthamoeba* keratitis in noncompliant soft contact lenses users: Genotyping and risk factors, a study from Cairo, Egypt. J. Infect. Public Health.

[46] Maghsood A.H., Sissons J., Rezaian M., Nolder D., Warhurst D., Khan N.A. (2005). Acanthamoeba genotype T4 from the UK and Iran and isolation of the T2 genotype from clinical isolates. J. Med Microbiol..

[47] Corsaro D., Walochnik J., Köhsler M., Rott M.B. (2015). *Acanthamoeba* misidentification and multiple labels: Redefining genotypes T16, T19, and T20 and proposal for *Acanthamoeba micheli* sp. nov.(genotype T19). Parasitol. Res..

[48] Fuerst P.A., Booton G.C., Crary M. (2015). Phylogenetic analysis and the evolution of the 18S rRNA gene typing system of *Acanthamoeba*. J. Eukaryot. Microbiol..

[49] Walochnik J., Obwaller A., Aspöck H. (2000). Correlations between morphological, molecular biological, and physiological characteristics in clinical and nonclinical isolates of *Acanthamoeba* spp.. Appl. Environ. Microbiol..

[50] Gomes T.d.S., Magnet A., Izquierdo F., Vaccaro L., Redondo F., Bueno S., Sánchez M.L., Angulo S., Fenoy S., Hurtado C. (2016). *Acanthamoeba* spp. in contact lenses from healthy individuals from Madrid, Spain. PLoS ONE.

[51] Walochnik J., Scheikl U., Haller-Schober E. (2015). Twenty years of *Acanthamoeba* diagnostics in Austria. J. Eukaryot. Microbiol..

[52] Casero R.D., Mongi F., Laconte L., Rivero F., Sastre D., Teherán A., Herrera G., Ramírez J.D. (2017). Molecular and morphological characterization of *Acanthamoeba* isolated from corneal scrapes and contact lens wearers in Argentina. Infect. Genet. Evol..

[53] Ledee D., Iovieno A., Miller D., Mandal N., Diaz M., Fell J., Fini M., Alfonso E.J. (2009). Molecular identification of T4 and T5 genotypes in isolates from *Acanthamoeba* keratitis patients. J. Clin. Microbiol..

[54] Maciver S.K., Asif M., Simmen M.W., Lorenzo-Morales J. (2013). A systematic analysis of *Acanthamoeba* genotype frequency correlated with source and pathogenicity: T4 is confirmed as a pathogen-rich genotype. Eur. J. Protistol..

[55] Di Cave D., Monno R., Bottalico P., Guerriero S., D’amelio S., D’orazi C., Berrilli F. (2009). *Acanthamoeba* T4 and T15 genotypes associated with keratitis infections in Italy. Eur. J. Clin. Microbiol. Infect. Dis..

[56] Roshni Prithiviraj S., Rajapandian S.G.K., Gnanam H., Gunasekaran R., Mariappan P., Sankalp Singh S., Prajna L. (2020). Clinical presentations, genotypic diversity and phylogenetic analysis of Acanthamoeba species causing keratitis. J. Med. Microbiol..

[57] Otero-Ruiz A., Gonzalez-Zuñiga L.D., Rodriguez-Anaya L.Z., Lares-Jiménez L.F., Gonzalez-Galaviz J.R., Lares-Villa F. (2022). Distribution and current state of molecular genetic characterization in pathogenic free-living amoebae. Pathogens.

[58] Ray D., Hayes R.E. (1954). *Hartmannella astronyxis*: A new species of free-living ameba. Cytology and life cycle. J. Morphol..

[59] Pussard M. (1964). *Acanthamoeba comandoni* n. sp., comparaison avec A. terricola. Rev. Ecol. Biol. Sol..

[60] Lewis E.J., Sawyer T.K. (1979). *Acanthamoeba tubiashi* n. sp., a new species of fresh-water Amoebida (Acanthamoebidae). Trans. Am. Microsc. Soc..

[61] Qvarnstrom Y., Nerad T.A., Visvesvara G.S. (2013). Characterization of a new pathogenic Acanthamoeba species, A. byersi n. sp., isolated from a human with fatal amoebic encephalitis. J. Eukaryot. Microbiol..

[62] Douglas M. (1930). Notes on the classification of the amoeba found by Castellani in cultures of a yeast-like fungus. J. Trop. Med. Hyg..

[63] Pussard M. (1964). Cytologie d’une Amibe terricola: *Acanthamoeba terricola* n. sp.. Ann. Sci. Nat. Zool..

[64] Schmoller H. (1964). Beschreibung einiger kulturamöben mariner herkunft. J. Protozool..

[65] Page F.C. (1967). Re-definition of the genus *Acanthamoeba* with descriptions of three species. J. Protozool..

[66] Sawyer T.K. (1971). *Acanthamoeba griffini*, a new species of marine amoeba. J. Protozool..

[67] Singh B.N., Das S.R. (1970). Studies on pathogenic and non-pathogenic small free-living amoebae and the bearing of nuclear division on the classification of the order Amoebida. Philos. Trans. R. Soc. Lond. B Biol. Sci..

[68] Sawyer T.K., Visvesvara G.S., Harke B.A. (1977). Pathogenic amoebas from brackish and ocean sediments, with a description of *Acanthamoeba hatchetti*, n. sp.. Science.

[69] Sawyer T.K., Nerad T.A., Lewis E.J., McLaughlin S.M. (1993). *Acanthamoeba stevensoni* N. Sp.(Protozoa: Amoebida) from Sewage-Contaminated Shellfish Beds in Raritan Bay, New York. Eukaryot. Microbiol..

[70] Nerad T.A., Sawyer T.K., Lewis E.J., McLaughlin S.M. (1995). *Acanthamoeba pearcei* n. sp. (Protozoa: Amoebida) from sewage contaminated sediments. J. Eukaryot. Microbiol..

[71] Tice A.K., Shadwick L.L., Fiore-Donno A.M., Geisen S., Kang S., Schuler G.A., Spiegel F.W., Wilkinson K.A., Bonkowski M., Dumack K. (2016). Expansion of the molecular and morphological diversity of Acanthamoebidae (Centramoebida, Amoebozoa) and identification of a novel life cycle type within the group. Biol. Direct.

[72] Reich K. (1933). Studien über die Bodenprotozoen Palästinas.

[73] Willaert E., Stevens A., Tyndall R.L. (1978). *Taxonomy Acanthamoeba royreba* sp. n. from a Human Tumor Cell Culture. J. Protozool..

[74] Molet B., Ermolieff-Braun G. (1976). Description d’Une Amibe d’Eau Douce: *Acanthamoeba lenticulata*, sp. nov. (Amoebida). Protistologica..

[75] Moura H., Wallace S., Visvesvara G.S. (1992). *Acanthamoeba healyi* n. sp. and the isoenzyme and immunoblot profiles of *Acanthamoeba* spp., groups 1 and 3. J. Protozool..

[76] Sawyer T.K., Nerad T.A., Visvesvara G.S. (1992). *Acanthamoeba jacobsi* sp. n.(Protozoa: Acanthamoebidae) from sewage contaminated ocean sediments. J. Helminthol. Soc. Wash..

[77] Im K., Shin H.J. (2003). *Acanthamoeba sohi*, n. sp., a pathogenic Korean isolate YM-4 from a freshwater fish. Korean J. Parasitol..

[78] Putaporntip C., Kuamsab N., Nuprasert W., Rojrung R., Pattanawong U., Tia T., Yanmanee S., Jongwutiwes S. (2021). Analysis of *Acanthamoeba* genotypes from public freshwater sources in Thailand reveals a new genotype, T23 *Acanthamoeba bangkokensis* sp. nov. Sci. Rep..

[79] Fanselow N., Sirajuddin N., Yin X.-T., Huang A.J., Stuart P.M. (2021). *Acanthamoeba* keratitis, pathology, diagnosis and treatment. Pathogens.

[80] Siddiqui R., Khan N.A. (2012). Biology and pathogenesis of *Acanthamoeba*. Parasites Vectors.

[81] Bowers B., Olszewski T. (1983). *Acanthamoeba* discriminates internally between digestible and indigestible particles. J. Cell Biol..

[82] Marciano-Cabral F., Cabral G. (2003). Acanthamoeba spp. as agents of disease in humans. Clin. Microbiol. Rev..

[83] Tan E.M., Starr M.R., Henry M.R., Pritt B.S. (2018). The brief case: A “fresh” pair of contact lenses. Am. Soc. Microbiol..

[84] Omaña-Molina M.A., González-Robles A., Salazar-Villatoro L., Bernal-Escobar A., Durán-Díaz Á., Méndez-Cruz A.R., Martínez-Palomo A.J.E., Lens C. (2014). Silicone hydrogel contact lenses surface promote Acanthamoeba castellanii trophozoites adherence: Qualitative and quantitative analysis. Eye Contact Lens.

[85] Weisman R.A. (1976). Differentiation in Acanthamoeba castellanii. Annu. Rev. Microbiol..

[86] Sriram R., Shoff M., Booton G., Fuerst P., Visvesvara G.S. (2008). Survival of Acanthamoeba cysts after desiccation for more than 20 years. J. Clin. Microbiol..

[87] Byers T.J., Akins R.A., Maynard B.J., Lefken R.A., Martin S.M. (1980). Rapid growth of Acanthamoeba in defined media; induction of encystment by glucose-acetate starvation. J. Protozool..

[88] Mazur T., Hadaś E., Iwanicka I. (1995). The duration of the cyst stage and the viability and virulence of Acanthamoeba isolates. Trop. Med. Parasitol. Off. Organ Dtsch. Tropenmedizinische Ges. Dtsch. Ges. Tech. Zusammenarbeit (GTZ).

[89] Bouheraoua N., Labbé A., Chaumeil C., Liang Q., Laroche L., Borderie V. (2014). Acanthamoeba keratitis. J. Fr. Ophtalmol..

[90] Lloyd D., Turner N., Khunkitti W., Hann A., Furr J., Russell A.D. (2001). Encystation in Acanthamoeba castellanii: Development of Biocide Resistance 1. J. Eukaryot. Microbiol..

[91] Turner N., Harris J., Russell A., Lloyd D. (2000). Microbial differentiation and changes in susceptibility to antimicrobial agents. J. Appl. Microbiol..

[92] Kremer I., Cohen E.J., Eagle R.C., Udell I., Laibson P.R. (1994). Histopathologic evaluation of stromal inflammation in Acanthamoeba keratitis. Eye Contact Lens.

[93] Kilvington S. (1993). *Acanthamoeba* trophozoite and cyst adherence to four types of soft contact lens and removal by cleaning agents. Eye.

[94] John T., Desai D., Sahm D. (1989). Adherence of *Acanthamoeba castellanii* cysts and trophozoites to unworn soft contact lenses. Arch. Ophthalmol..

[95] Niederkorn J.Y. (2021). The biology of Acanthamoeba keratitis. Exp. Eye Res..

[96] Omaña-Molina M., Hernandez-Martinez D., Sanchez-Rocha R., Cardenas-Lemus U., Salinas-Lara C., Mendez-Cruz A.R., Colin-Barenque L., Aley-Medina P., Espinosa-Villanueva J., Moreno-Fierros L. (2017). In vivo CNS infection model of *Acanthamoeba* genotype T4: The early stages of infection lack presence of host inflammatory response and are a slow and contact-dependent process. Parasitol. Res..

[97] Huth S., Reverey J.F., Leippe M., Selhuber-Unkel C. (2017). Adhesion forces and mechanics in mannose-mediated acanthamoeba interactions. PLoS ONE.

[98] Yoo K.-T., Jung S.-Y. (2012). Effects of mannose on pathogenesis of *Acanthamoeba castellanii*. Korean J. Parasitol..

[99] Sharma C., Khurana S., Bhatia A., Arora A., Gupta A. (2023). The gene expression and proteomic profiling of Acanthamoeba isolates. Exp. Parasitol..

[100] Matin A., Jung S.-Y. (2011). Phospholipase activities in clinical and environmental isolates of *Acanthamoeba*. Korean J. Parasitol..

[101] Lorenzo-Morales J., Khan N.A., Walochnik J. (2015). An update on *Acanthamoeba* keratitis: Diagnosis, pathogenesis and treatment. Parasite.

[102] Pellegrin J., Ortega-Barria E., Barza M., Baum J., Pereira M. (1991). Neuraminidase activity in acanthamoeba species trophozoites and cysts. Investig. Ophthalmol. Vis. Sci..

[103] Panjwani N. (2010). Pathogenesis of *Acanthamoeba* keratitis. Ocul. Surf..

[104] Alves D.d.S.M.M., Gonçalves G.S., Moraes A.S., Alves L.M., Carmo Neto J.R.d., Hecht M.M., Nitz N., Gurgel-Gonçalves R., Bernardes G., de Castro A.M. (2018). The first *Acanthamoeba* keratitis case in the Midwest region of Brazil: Diagnosis, genotyping of the parasite and disease outcome. Rev. Soc. Bras. Med. Trop..

[105] Serrano-Luna J.d.J., Cervantes-Sandoval I., Calderón J., Navarro-García F., Tsutsumi V., Shibayama M. (2006). Protease activities of Acanthamoeba polyphaga and *Acanthamoeba castellanii*. Can. J. Microbiol..

[106] Cirelli C., Mesquita E.I.S., Chagas I.A.R., Furst C., Possamai C.O., Abrahão J.S., dos Santos Silva L.K., Grossi M.F., Tagliati C.A., Costa A.O. (2020). Extracellular protease profile of *Acanthamoeba* after prolonged axenic culture and after interaction with MDCK cells. Parasitol. Res..

[107] Moon E.-K., Hong Y., Chung D.-I., Kong H.-H. (2012). Cysteine protease involving in autophagosomal degradation of mitochondria during encystation of Acanthamoeba. Mol. Biochem. Parasitol..

[108] Hong Y., Kang J.-M., Joo S.-Y., Song S.-M., Lê H.G., Thái T.L., Lee J., Goo Y.-K., Chung D.-I., Sohn W.-M. (2018). Molecular and biochemical properties of a cysteine protease of Acanthamoeba castellanii. Korean J. Parasitol..

[109] Ramírez-Rico G., Martínez-Castillo M., De La Garza M., Shibayama M., Serrano-Luna J. (2015). *Acanthamoeba castellanii* proteases are capable of degrading iron-binding proteins as a possible mechanism of pathogenicity. J. Eukaryot. Microbiol..

[110] Łanocha-Arendarczyk N., Baranowska-Bosiacka I., Gutowska I., Kolasa-Wołosiuk A., Kot K., Łanocha A., Metryka E., Wiszniewska B., Chlubek D., Kosik-Bogacka D. (2018). The activity of matrix metalloproteinases (MMP-2, MMP-9) and their tissue inhibitors (TIMP-1, TIMP-3) in the cerebral cortex and hippocampus in experimental acanthamoebiasis. Int. J. Mol. Sci..

[111] Sissons J., Alsam S., Goldsworthy G., Lightfoot M., Jarroll E.L., Khan N.A. (2006). Identification and properties of proteases from an Acanthamoeba isolate capable of producing granulomatous encephalitis. BMC Microbiol..

[112] Van Klink F., Taylor W.M., Alizadeh H., Jager M.J., Van Rooijen N., Niederkorn J.Y. (1996). The role of macrophages in Acanthamoeba keratitis. Investig. Ophthalmol. Vis. Sci..

[113] Marciano-Cabral F., Toney D.M. (1998). The interaction of *Acanthamoeba* spp. with activated macrophages and with macrophage cell lines. J. Eukaryot. Microbiol..

[114] Hurt M., Proy V., Niederkorn J.Y., Alizadeh H. (2003). The interaction of Acanthamoeba castellanii cysts with macrophages and neutrophils. J. Parasitol..

[115] Stewart G., Shupe K., Kim I., Silvany R., Alizadeh H., McCulley J.P., Niederkorn J.Y. (1994). Antibody-dependent neutrophil-mediated killing of *Acanthamoeba castellanii*. Int. J. Parasitol..

[116] Clarke D.W., Alizadeh H., Niederkorn J.Y. (2005). Failure of *Acanthamoeba castellanii* to produce intraocular infections. Investig. Ophthalmol. Vis. Sci..

[117] Cano A., Mattana A., Woods S., Henriquez F.L., Alexander J., Roberts C.W. (2017). Acanthamoeba activates macrophages predominantly through Toll-like receptor 4-and MyD88-dependent mechanisms to induce interleukin-12 (IL-12) and IL-6. Infect. Immun..

[118] Carnt N., Montanez V.M., Galatowicz G., Veli N., Calder V. (2017). Tear cytokine levels in contact lens wearers with acanthamoeba keratitis. Cornea.

[119] Carnt N.A., Pang I., Burdon K.P., Calder V., Dart J.K., Subedi D., Hardcastle A.J. (2021). Innate and adaptive gene single nucleotide polymorphisms associated with susceptibility of severe inflammatory complications in *Acanthamoeba* keratitis. Investig. Ophthalmol. Vis. Sci..

[120] Alizadeh H., Apte S., El-Agha M.-S.H., Li L., Hurt M., Howard K., Cavanagh H.D., McCulley J.P., Niederkorn J. (2001). Tear IgA and serum IgG antibodies against *Acanthamoeba* in patients with *Acanthamoeba* keratitis. Cornea.

[121] Feng X., Zheng W., Wang Y., Zhao D., Jiang X., Lv S. (2015). A rabbit model of *Acanthamoeba* keratitis that better reflects the natural human infection. Anat. Rec. Adv. Integr. Anat. Evol. Biol..

[122] Pumidonming W., Walochnik J., Dauber E., Petry F. (2011). Binding to complement factors and activation of the alternative pathway by *Acanthamoeba*. Immunobiology.

[123] Alizadeh H., He Y., McCulley J.P., Ma D., Stewart G.L., Via M., Haehling E., Niederkorn J.Y. (1995). Successful immunization against *Acanthamoeba* keratitis in a pig model. Cornea.

[124] Kong H.-H., Kim T.-H., Chung D.-I. (2000). Purification and characterization of a secretory serine proteinase of Acanthamoeba healyi isolated from GAE. J. Parasitol..

[125] Foulks G.N. (2007). Acanthamoeba keratitis and contact lens wear: Static or increasing problem?. Eye Contact Lens..

[126] Neelam S., Niederkorn J.Y. (2017). Focus: Infectious diseases: Pathobiology and immunobiology of Acanthamoeba keratitis: Insights from animal models. Yale J. Biol. Med..

[127] Mathers W., Stevens G., Rodrigues M., Chan C.C., Gold J., Visvesvara G.S., Lemp M.A., Zimmerman L.E. (1987). Immunopathology and electron microscopy of *Acanthamoeba* keratitis. Am. J. Ophthalmol..

[128] Clarke D.W., Niederkorn J.Y. (2006). The immunobiology of *Acanthamoeba* keratitis. Microbes Infect..

[129] Klink F.V., Leher H., Jager M., Alizadeh H., Taylor W., Niederkorn J.Y. (1997). Systemic immune response to *Acanthamoeba* keratitis in the Chinese hamster. Ocul. Immunol. Inflamm..

[130] Huang J.-M., Chang Y.-T., Shih M.-H., Lin W.-C., Huang F.-C. (2019). Identification and characterization of a secreted M28 aminopeptidase protein in *Acanthamoeba*. Parasitol. Res..

[131] Betanzos A., Bañuelos C., Orozco E. (2019). Host invasion by pathogenic amoebae: Epithelial disruption by parasite proteins. Genes.

[132] Willcox M.D.P. (2019). Tear film, contact lenses and tear biomarkers. Clin. Exp. Optom..

[133] Ismail N.N., Yusof H. (2021). Occurrence of The Pathogenic Amoeba *Naegleria fowleri*, Pathogenesis, Diagnosis, and Treatment Options. Malays. J. Med. Health Sci..

[134] Knickelbein J.E., Kovarik J., Dhaliwal D.K., Chu C.T. (2013). *Acanthamoeba* keratitis: A clinicopathologic case report and review of the literature. Hum. Pathol..

[135] Niederkorn J.Y. (2008). Innate and adaptive immune responses to ocular Acanthamoeba infections. Expert Rev. Ophthalmol..

[136] McClellan K., Howard K., Mayhew E., Niederkorn J.Y., Alizadeh H. (2002). Adaptive immune responses to *Acanthamoeba* cysts. Exp. Eye Res..

[137] Carnt N.A., Willcox M.D., Hau S., Garthwaite L.L., Evans V.E., Radford C.F., Dart J.K., Chakrabarti S., Stapleton F. (2012). Association of single nucleotide polymorphisms of interleukins-1β,-6, and-12B with contact lens keratitis susceptibility and severity. Ophthalmology.

[138] Carnt N.A., Willcox M.D., Hau S., Keay L., Dart J.K., Chakrabarti S., Stapleton F. (2012). Immune defense single nucleotide polymorphisms and recruitment strategies associated with contact lens keratitis. Ophthalmology.

[139] Carnt N.A., Cipriani V., Stapleton F.J., Calder V., Willcox M.D. (2019). Association study of single nucleotide polymorphisms in IL-10 and IL-17 genes with the severity of microbial keratitis. Contact Lens Anterior Eye.

[140] Megha K., Sharma M., Gupta A., Sehgal R., Khurana S. (2021). Microbiological diagnosis of *Acanthamoebic* keratitis: Experience from tertiary care center of North India. Diagn. Microbiol. Infect. Dis..

[141] Szentmáry N., Daas L., Shi L., Laurik K.L., Lepper S., Milioti G., Seitz B. (2019). Acanthamoeba keratitis—Clinical signs, differential diagnosis and treatment. J. Curr. Ophthalmol..

[142] Yera H., Ok V., Kuet F.L.K., Dahane N., Ariey F., Hasseine L., Delaunay P., Martiano D., Marty P., Bourges J.L. (2021). PCR and culture for diagnosis of *Acanthamoeba* keratitis. Br. J. Ophthalmol..

[143] Goh J.W., Harrison R., Hau S., Alexander C.L., Tole D.M., Avadhanam V.S.M. (2018). Comparison of in vivo confocal microscopy, PCR and culture of corneal scrapes in the diagnosis of *Acanthamoeba* keratitis. Cornea.

[144] Penland R.L., Wilhelmus K.R. (1997). Comparison of axenic and monoxenic media for isolation of Acanthamoeba. J. Clin. Microbiol..

[145] Muiño L., Rodrigo D., Villegas R., Romero P., Peredo D.E., Vargas R.A., Liempi D., Osuna A., Jercic M.I. (2019). Effectiveness of sampling methods employed for *Acanthamoeba* keratitis diagnosis by culture. Int. Ophthalmol..

[146] Grossniklaus H.E., Waring IV G.O., Akor C., Castellano-Sanchez A.A., Bennett K. (2003). Evaluation of hematoxylin and eosin and special stains for the detection of acanthamoeba keratitis in penetrating keratoplasties. Am. J. Ophthalmol..

[147] Kheirkhah A., Satitpitakul V., Syed Z.A., Müller R., Goyal S., Tu E.Y., Dana R. (2018). Factors influencing the diagnostic accuracy of laser-scanning in vivo confocal microscopy for *Acanthamoeba* keratitis. Cornea.

[148] De Craene S., Knoeri J., Georgeon C., Kestelyn P., Borderie V.M. (2018). Assessment of confocal microscopy for the diagnosis of polymerase chain reaction–positive *Acanthamoeba* keratitis: A case-control study. Ophthalmology.

[149] Alkatan H.M., Al-Essa R.S. (2019). Challenges in the diagnosis of microbial keratitis: A detailed review with update and general guidelines. Saudi J. Ophthalmol..

[150] Li S., Bian J., Wang Y., Wang S., Wang X., Shi W. (2020). Clinical features and serial changes of Acanthamoeba keratitis: An in vivo confocal microscopy study. Eye.

[151] Lehmann O.J., Green S.M., Morlet N., Kilvington S., Keys M.F., Matheson M.M., Dart J., McGill J.I., Watt P.J. (1998). Polymerase chain reaction analysis of corneal epithelial and tear samples in the diagnosis of *Acanthamoeba* keratitis. Invest. Ophthalmol. Vis. Sci..

